# Efficacy and safety of transoral endoscopic thyroidectomy vestibular approach of papillary thyroid carcinoma: a network meta-analysis

**DOI:** 10.3389/fonc.2026.1869610

**Published:** 2026-07-01

**Authors:** Pengyu Li, Kai Li

**Affiliations:** Beijing Tiantan Hospital, Capital Medical University, Beijing, China

**Keywords:** axillary breast approach, endoscopic thyroidectomy, network meta-analysis, open thyroidectomy, papillary thyroid carcinoma, transoral endoscopic thyroidectomy vestibular approach

## Abstract

**Background:**

This network meta-analysis aimed to systematically evaluate the efficacy and safety of the transoral endoscopic thyroidectomy vestibular approach (TOETVA) compared with open thyroidectomy (OT) and the endoscopic thyroidectomy via axillary breast approach (ABA) for treating papillary thyroid carcinoma (PTC).

**Materials and methods:**

Five databases (PubMed, Embase, Web of Science, Cochrane Library, and Scopus) were systematically searched from inception to January 31, 2026, for clinical studies comparing at least two of the three thyroidectomy approaches (TOETVA, OT, ABA) in patients with PTC. A network meta-analysis was conducted using RStudio (version 2025.05.1). Standardized mean differences and odds ratios with 95% confidence intervals were calculated. Statistical significance was set at P < 0.05.

**Results:**

A total of 28 studies involving 8,047 patients were included. In this cohort, 1,623 patients underwent TOETVA, 5,605 underwent OT, and 819 underwent ABA. The network meta-analysis revealed the following findings: (1) Surgical efficiency: Both OT and ABA were associated with shorter operative times than TOETVA (MD = −73.85 minutes, P < 0.001; MD = −30.59 minutes, P < 0.001). (2) Postoperative recovery: OT was superior to TOETVA in terms of shorter postoperative hospital stay (MD = −0.49 days, P = 0.042) and lower drainage volume (MD = −58.03 mL, P = 0.012), while no significant differences were observed between ABA and TOETVA for these two outcomes. (3) Tumor radicality: No statistically significant differences were found among the three approaches regarding the number of lymph nodes dissected, the number of metastatic lymph nodes, or postoperative recurrence rate. (4) Nerve protection: No differences were observed among the three approaches in terms of temporary or permanent recurrent laryngeal nerve (RLN) injury. However, OT was superior to TOETVA in sensory nerve protection (OR = 0.09, P = 0.010). (5) Parathyroid function: TOETVA was superior to OT in preventing temporary hypoparathyroidism (OR = 0.48, P = 0.023). (6) Other complications: No significant differences were found among the three approaches in terms of bleeding, hematoma, seroma, or infection.

**Conclusions:**

TOETVA, OT, and ABA all demonstrated safe and reliable clinical efficacy for PTC. TOETVA represents a safe and feasible alternative to OT, offering comparable surgical and oncological outcomes with the added benefits of better parathyroid protection.

## 1 Introduction

The global incidence of thyroid cancer (TC) has been steadily rising (1). Papillary thyroid carcinoma (PTC) is the predominant histological subtype, accounting for over 80% of TC cases (2). PTC generally carries a favorable prognosis, with 10-year survival rates exceeding 90% (3). Surgical resection is the primary treatment for PTC, and traditional open thyroidectomy (OT) has long been regarded as the standard treatment modality (4). However, OT inevitably leaves a visible cervical scar, which can adversely affect patients’ body image and cause psychological distress (5). In response to the growing emphasis on aesthetics and the development of minimally invasive techniques, endoscopic thyroid surgery has emerged, leading to the development of various endoscopic thyroidectomy approaches, including trans-mammary, full-areolar, axillary, axillo-mammary, and transoral, among others (6).

Among these, the transoral endoscopic thyroidectomy vestibular approach (TOETVA) has gained significant traction in recent years. As a natural orifice transluminal endoscopic surgery (NOTES) procedure, TOETVA offers theoretical advantages, including the absence of visible scarring, excellent cosmetic outcomes, and a short subcutaneous working distance (7, 8). The axillary breast approach (ABA) is another commonly employed endoscopic thyroidectomy technique (9). While ABA does not achieve a completely scarless result, it provides aesthetic benefits by concealing the incision in less visible areas (10).

Despite its growing adoption, the efficacy and safety of TOETVA for PTC remain subjects of debate. Although several studies have compared surgical outcomes between TOETVA and OT, or between TOETVA and ABA, the conclusions have been inconsistent (11–14). With the widespread clinical application of TOETVA, a comprehensive, high-level synthesis of evidence is urgently needed. To date, no network meta-analysis has directly compared all three approaches—TOETVA, OT, and ABA—in a single analytical framework. This study represents the first network meta-analysis to systematically compare these three techniques for PTC across multiple domains, including surgical efficiency, postoperative recovery, tumor radicality, complications, and recurrence. The aim is to characterize the efficacy and safety of TOETVA in the treatment of PTC more clearly, thereby providing evidence-based guidance for clinical decision-making.

## 2 Materials and methods

### 2.1 Search strategy

This network meta-analysis was conducted according to the Preferred Reporting Items for Systematic Reviews and Meta-Analyses (PRISMA) 2020 guidelines (15). Five electronic databases (PubMed, Embase, Web of Science, Cochrane Library, Scopus) were systematically searched for literature published up to January 31, 2026, using the following search strategy: (“Thyroid Cancer, Papillary” OR “Thyroid Neoplasms” OR “papillary thyroid carcinomas” OR “papillary thyroid carcinoma” OR Papillary “Thyroid Cancers” OR “papillary thyroid cancer” OR “PTC”) AND (“Thyroidectomy” OR “thyroidectomy) AND (“Endoscopic Surgery” OR “Natural Orifice Endoscopic Surgery” OR “minimally invasive” OR “scarless”) AND (“transoral” OR “oral vestibular” OR “transoral endoscopic thyroidectomy” OR “TOETVA” OR “transoral vestibular” OR “axillo-breast” OR “bilateral axillo-breast” OR “BABA” OR “conventional” OR “open” OR “open thyroidectomy” OR “traditional” OR “transcervical”). Eligible studies directly compared TOETVA with ABA, or either TOETVA or ABA with OT. Only human studies published in English with full-text descriptions were considered. Two independent reviewers determined final inclusion of studies, and a third author resolved any discrepancies.

### 2.2 Inclusion and exclusion criteria

Inclusion criteria: 1) Patients diagnosed with papillary thyroid carcinoma, with no age restrictions. 2) Patients in the intervention group underwent TOETVA. 3) Patients in the control group underwent ABA or OT. 4) At least one of the following outcomes reported: operative time, intraoperative blood loss volume, postoperative hospital stay in days, postoperative drainage volume, the number of retrieved lymph nodes, the number of metastatic lymph nodes, incidences of transient/permanent RLN palsy, transient/permanent hypoparathyroidism, postoperative blood loss, hematoma, seroma, infection, sensory nerve injury, or recurrence.

Exclusion criteria: 1) Other types of articles, such as case reports, abstracts, publications, letters, reviews, meta-analyses, editorials, animal studies, and protocols; 2) Not relevant; 3) Other diseases, such as benign thyroid lesions or other histological types of thyroid cancer; 4) Full text unavailable; 5) Published non-English; 6) Reduplicated cohort of patients; 7) Failed to extract data.

### 2.3 Data extraction and quality assessment

Two reviewers independently performed literature screening, data extraction, and cross-checking. The extracted data included the first author, publication year, study period, country, study design, surgical approach, sample size, patient age, patient sex, and type of surgery. The quality of studies was evaluated using the Risk of Bias in Non-randomized Studies of Interventions (ROBINS-I) tool.

### 2.4 Statistical analysis

Network meta-analysis was performed using the gemtc, netmeta, and meta packages in RStudio (version 2025.05.1). Continuous variables were expressed as mean difference (MD) with 95% confidence interval (CI), while dichotomous variables were expressed as odds ratio (OR) with 95% CI.

Statistical heterogeneity was assessed using the I² statistic. An I² value > 50% indicated substantial heterogeneity, in which case a random-effects model was adopted; otherwise, a fixed-effects model was used. Consistency was evaluated using node-splitting analysis. Treatment rankings were determined using the Surface Under the Cumulative Ranking (SUCRA) values. Convergence of the Bayesian models was assessed using Gelman-Rubin diagnostic plots and trace plots. A P value < 0.05 was considered statistically significant.

## 3 Results

### 3.1 Search results

**Figure 1:**
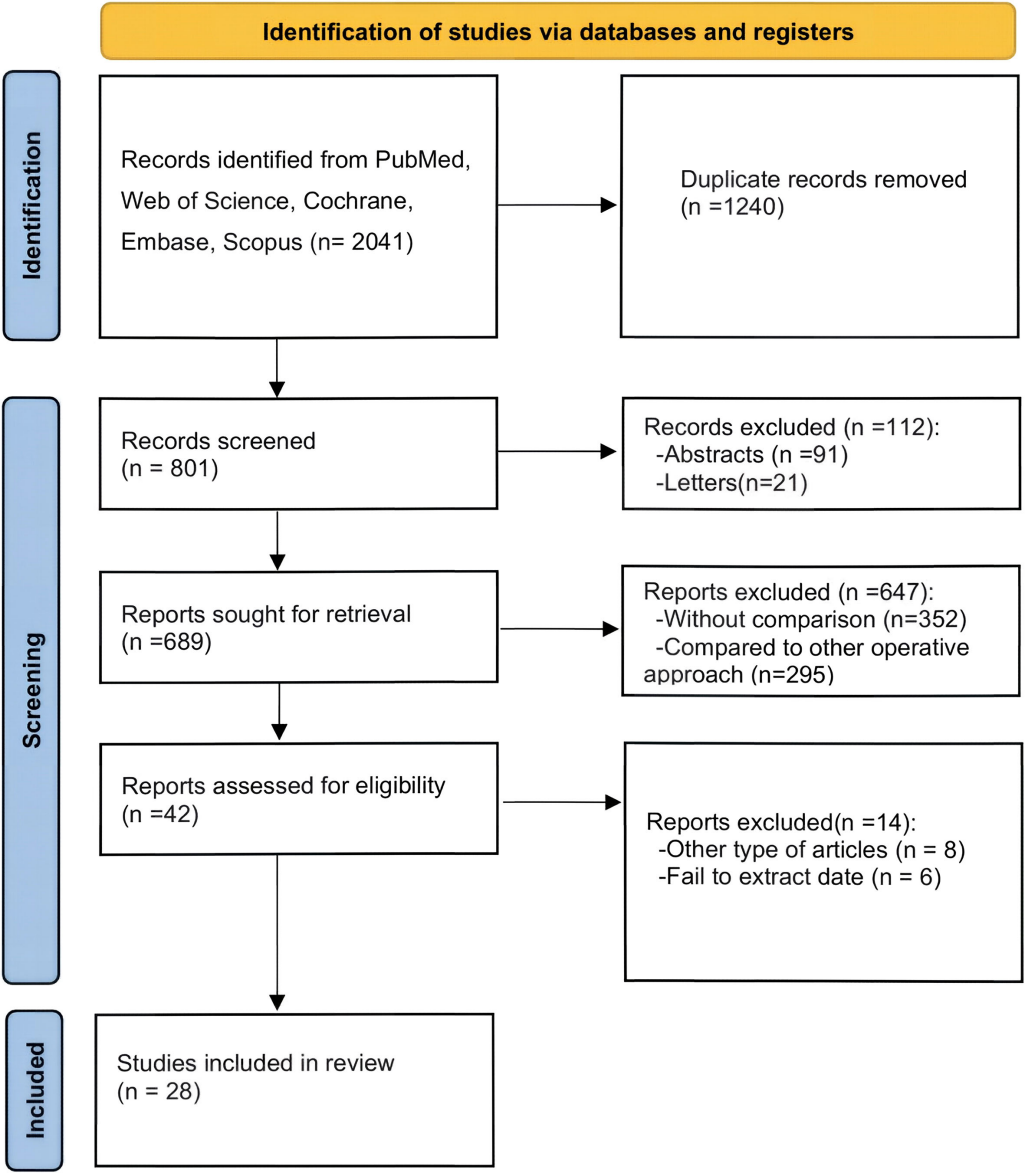
PRISMA flow diagram shows the process of the study selection.

The process of literature selection and inclusion is presented in **Figure 1**. The initial search identified 2,041 studies. After deduplication, 801 remained. Following abstract screening, 112 were excluded, leaving 689 eligible articles. Full-text review led to the exclusion of 647 additional articles. Ultimately, 28 studies (17 retrospective cohort, 11 prospective cohort) were included.

### 3.2 Study characteristics

This meta-analysis included a total of 28 studies. The distribution of pairwise comparisons among the three thyroidectomy approaches was as follows: OT versus TOETVA (16 studies), ABA versus TOETVA (4 studies), and ABA versus OT (8 studies). A total of 8,047 patients were included, of whom 1,623 underwent TOETVA, 5,605 underwent OT, and 819 underwent ABA. The key characteristics of each included study are summarized in **Table 1**. The results of the quality assessment using the ROBINS-I tool are presented in **Supplementary Figure 1**. The network relationships among the different surgical approaches for each outcome are illustrated in **Supplementary Figure 2**.

**Table 1:** Characteristics of the 28 included studies.

Author	Year	Study period	Country	Study design	Approach	sample of patients	Age (years) mean± SD	Sex (F/M)	Type of surgery (TT/Lobectomy)
Xu et al. (16)	2026	2022.6-2024.10	China	Prospective cohort study	TOETVA	108	34.02 ± 6.10	108/0	0/108
					OT	128	33.08 ± 5.52	128/0	0/128
Lee et al. (17)	2025	2016-2023	Korea	Retrospective cohort study	TOETVA	128	43.1 ± 8.1	105/19	6/118
					OT	559	43.0 ± 8.9	98/26	9/115
Zhang et al. (18)	2025	2019.10-2023.12	China	Retrospective cohort study	TOETVA	59	32.0 ± 8.1	56/3	0/59
					OT	324	37.0 ± 11.5	215/109	0/324
Li et al. (19)	2025	2019.9-2020.1	China	Retrospective cohort study	TOETVA	80	37.0 ± 10.0	73/7	17/63
					OT	30	47.4 ± 7.9	20/10	13/17
Barczyński et al. (20)	2025	2020-2023	Poland	Prospective cohort study	TOETVA	17	41.2 ± 6.7	15/2	NA
					OT	23	53.2 ± 7.6	20/3	NA
Wu et al. (21)	2025	2017.1-2024.9	China	Retrospective cohort study	TOETVA	85	38.39 ± 10.26	51/34	13/72
					OT	136	47.85 ± 12.02	94/42	52/84
Park et al. (22)	2024	2017.10-2022.2	Korea	Prospective cohort study	TOETVA	310	43.6 ± 12.3	145/49	32/162
					OT	194	54.1 ± 14.4	213/97	121/189
Li et al. (23)	2023	2016.4-2021.12	China	Retrospective cohort study	TOETVA	107	31.7 ± 7.0	101/6	107/0
					OT	673	42.8 ± 9.2	480/193	673/0
Bhandarwar et al. (24)	2023	2015.1-2020.12	India	Retrospective cohort study	TOETVA	47	35.8 ± 4.07	42/5	39/8
					ABA	72	34.21 ± 3.74	60/12	0/72
Sun et al. (25)	2022	2017.6-2021.10	China	Retrospective cohort study	TOETVA	28	36.57 ± 8.03	27/1	28/0
					OT	56	39.66 ± 8.67	54/2	56/0
Wongwattana et al. (26)	2021	2017.8-2018.7	Thailand	Prospective cohort study	TOETVA	11	39.27 ± 10.19	11/0	0/11
					ABA	11	43.36 ± 10.74	11/0	0/111
Liang et al. (27)	2021	2018.10-2020.8	Taiwan	Retrospective cohort study	TOETVA	72	44.7 ± 12.0	64/8	14/58
					ABA	63	49.3 ± 11.3	57/6	20/43
Nguyen et al. (28)	2021	2018-2019	Vietnam	Prospective cohort study	TOETVA	51	45.1 ± 11.8	46/5	40/11
					ABA	50	34.5 ± 8.4	48/2	0/50
Hong et al. (29)	2020	2018.1-2019.4	Korea	Retrospective cohort study	TOETVA	82	44.2 ± 12.3	79/3	12/70
					OT	233	47.8 ± 12(	186/47	77/156
Wang et al. (30)	2020	2015.6-2016.9	China	Prospective cohort study	TOETVA	80	31.48 ± 6.60	80/0	7/73
					OT	80	32.59 ± 5.18	80/0	36/44
Ahn et al. (31)	2020	2016.8-2019.3	Korea	Retrospective cohort study	TOETVA	150	43.06 ± 10.90	145/5	40/110
					OT	125	51.02 ± 12.42	89/36	85/40
Kasemsiri et al. (32)	2020	2017.1-2018.11	Thailand	Prospective cohort study	TOETVA	32	38.3 ± 11.3	32/0	0/32
					OT	38	46.7 ± 10.9	34/4	0/38
Sun et al. (6)	2020	2017.1-2018.6	China	Retrospective cohort study	TOETVA	100	29.65 ± 6.57	86/14	0/100
					OT	289	45.18 ± 11.47	165/124	0/289
Pérez-Soto et al. (33)	2019	2017.7-2019.4	Mexico	Retrospective cohort study	TOETVA	20	48.10 ± 15.67	NA	14/6
					OT	20	45.55 ± 14.42	NA	16/4
Weng et al. (34)	2025	2021.1-2023.12	China	Retrospective cohort study	OT	350	35.97 ± 8.16	320/30	55/295
					ABA	83	30.86 ± 6.77	75/8	3/80
Saavedra-Pérez et al. (35)	2023	2017.8-2020.8	Spain	Prospective cohort study	OT	100	43± 11.5	86/14	0/100
					ABA	100	42± 8.1	89/11	0/100
Wirth et al. (36)	2021	NA	Germany	Prospective cohort study	OT	225	59.0 ± 12.2	151/74	151/74
					ABA	52	48.4 ± 12.0	52/0	42/10
Johri et al. (37)	2020	2016.10-2018.8	India	Prospective cohort study	OT	52	33.0 ± 9.6	39/13	0/52
					ABA	41	30.7 ± 9.2	37/4	0/41
Alramadhan et al. (38)	2017	2008-2015	Korea	Retrospective cohort study	OT	262	50.7 ± 12.9	183/79	77/185
					ABA	95	36.5 ± 9.8	93/2	11/84
Park et al. (39)	2016	2011.1-2011.12	Korea	Prospective cohort study	OT	102	50.8 ± 11.5	88/14	102/0
					ABA	50	38.0 ± 9.4	46/4	50/0
Kim et al. (40)	2016	2007.5-2011.2	Korea	Retrospective cohort study	OT	830	49.53 ± 16.75	734/96	689/141
					ABA	173	38.90 ± 10.00	160/13	57/116
Koh et al. (41)	2010	2006.1-2007.12	Korea	Retrospective cohort study	OT	30	38.3 ± 4.5	24/6	6/24
					ABA	29	36.5 ± 5.1	26/3	4/25
Lira et al. (42)	2020	2018.1-2019.5	Brazil	Retrospective cohort study	TOETVA	56	40.8 ± 12.25	49/7	37/19
					OT	745	45.2 ± 20.00	606/140	614/132

Data are presented as mean ± SD.

N/A, not available; OT, Open thyroidectomy; TOETVA, Transoral endoscopic thyroidectomy vestibular approach; ABA, endoscopic thyroidectomy via the Axillary Breast Approach; TT, total thyroidectomy; F/M, female/male.

### 3.3 Outcomes

A summary of the meta-analysis results for all clinical outcomes is presented in **Table 2**.

**Table 2:** Summary of network meta-analysis results for all outcomes.

Outcomes	No. of studies	Group	Heterogeneity		Overall effect size	95% CI of overall effect		P Value
			I ^2^(%)	P Value				
Operative time (mins)	26	TOETVA vs OT	97.4	<0.001	-73.85		(-86.54, -61.16)	<0.001
		TOETVA vs ABA			-30.59		(-47.75, -13.43)	0.0005
		OT vs ABA			43.26		(27.78, 58.75)	<0.001
Intraoperative blood loss (mL)	12	TOETVA vs OT	86	<0.001	-0.49		(-0.96, -0.02)	0.042
		TOETVA vs ABA			0.05		(-0.55, 0.65)	0.878
		OT vs ABA			0.54		(-0.02, 1.09)	0.062
Postoperative hospital stay (days)	22	TOETVA vs OT	96.8	<0.001	-0.49		(-0.96, -0.02)	0.042
		TOETVA vs ABA			0.05		(-0.55, 0.65)	0.878
		OT vs ABA			0.54		(-0.02, 1.09)	0.062
Postoperative drainage (ml)	10	TOETVA vs OT	98.1	<0.001	-58.03		(-103.14, -12.92)	0.012
		TOETVA vs ABA			32.04		(-24.59, 88.66)	0.268
		OT vs ABA			90.06		(36.69, 143.44)	<0.001
Number of retrieved central lymph nodes (CLN)	15	TOETVA vs OT	97.5	<0001	1.01		(-0.96, 2.98)	0.313
		TOETVA vs ABA			-1.73		(-4.99, 1.53)	0.297
		OT vs ABA			-2.75		(-5.63, 0.14)	0.062
Number of metastatic lymph nodes	13	TOETVA vs OT	50.9	0.021	-0.05		(-0.26, 0.17)	0.674
		TOETVA vs ABA			-0.14		(-0.56, 0.28)	0.515
		OT vs ABA			-0.10		(-0.46, 0.27)	0.604
Transient RLN palsy	23	TOETVA vs OT	0	0.80	1.06		(0.72, 1.56)	0.780
		TOETVA vs ABA			1.29		(0.75, 2.20)	0.355
		OT vs ABA			1.22		(0.78, 1.89)	0.378
Permanent RLN palsy	7	TOETVA vs OT	0	0.97	1.57		(0.56, 4.39)	0.392
		TOETVA vs ABA			1.67		(0.41, 6.87)	0.476
		OT vs ABA			1.07		(0.41, 2.81)	0.378
Transient hypoparathyroidism	20	TOETVA vs OT	74.7	<0.001	0.48		(0.26, 0.90)	0.023
		TOETVA vs ABA			1.37		(0.54, 3.48)	0.512
		OT vs ABA			0.66		(0.28, 1.58)	0.378
Permanent hypoparathyroidism	8	TOETVA vs OT	0	0.97	1.57		(0.56, 4.39)	0.392
		TOETVA vs ABA			1.67		(0.41, 6.87)	0.476
		OT vs ABA			1.07		(0.41, 2.81)	0.378
Postoperative blood loss	4	TOETVA vs OT	0	0.37	1.28		(0.14, 11.71)	0.824
		TOETVA vs ABA			0.90		(0.05, 16.91)	0.941
		OT vs ABA			0.70		(0.10, 4.83)	0.378
Hematoma	9	TOETVA vs OT	58.6	0.018	0.87		(0.13, 5.79)	0.889
		TOETVA vs ABA			2.36		(0.19, 29.85)	0.508
		OT vs ABA			2.70		(0.30, 24.16)	0.378
Seroma	6	TOETVA vs OT	0	0.48	0.55		(0.19, 1.64)	0.285
		TOETVA vs ABA			0.56		(0.10, 3.12)	0.511
		OT vs ABA			1.02		(0.22, 4.79)	0.378
Infection	8	TOETVA vs OT	0	0.82	0.36		(0.12, 1.05)	0.060
		TOETVA vs ABA			0.86		(0.08, 9.26)	0.904
		OT vs ABA			2.42		(0.23, 25.77)	0.378
Sensory nerve injury	7	OT vs TOETVA	49.3	0.079	0.09		(0.01, 0.56)	0.010
		ABA vs TOETVA		1.29		(0.23, 7.14)	0.771
		OT vs ABA		14.17		(1.55, 129.18)	0.016
Recurrence	3	TOETVA vs OT	0	0.47	2.93		(0.12, 72.95)	0.512
		TOETVA vs ABA	2.92		(0.10, 88.87)	0.539
		OT vs ABA	1.00		(0.31, 3.16)	–

TOETVA, transoral endoscopic thyroidectomy vestibular approach; ABA, endoscopic thyroidectomy via the Axillary Breast Approach; WMD, weighted mean difference.

#### 3.3.1 Intraoperative outcomes

**Figure 2:**
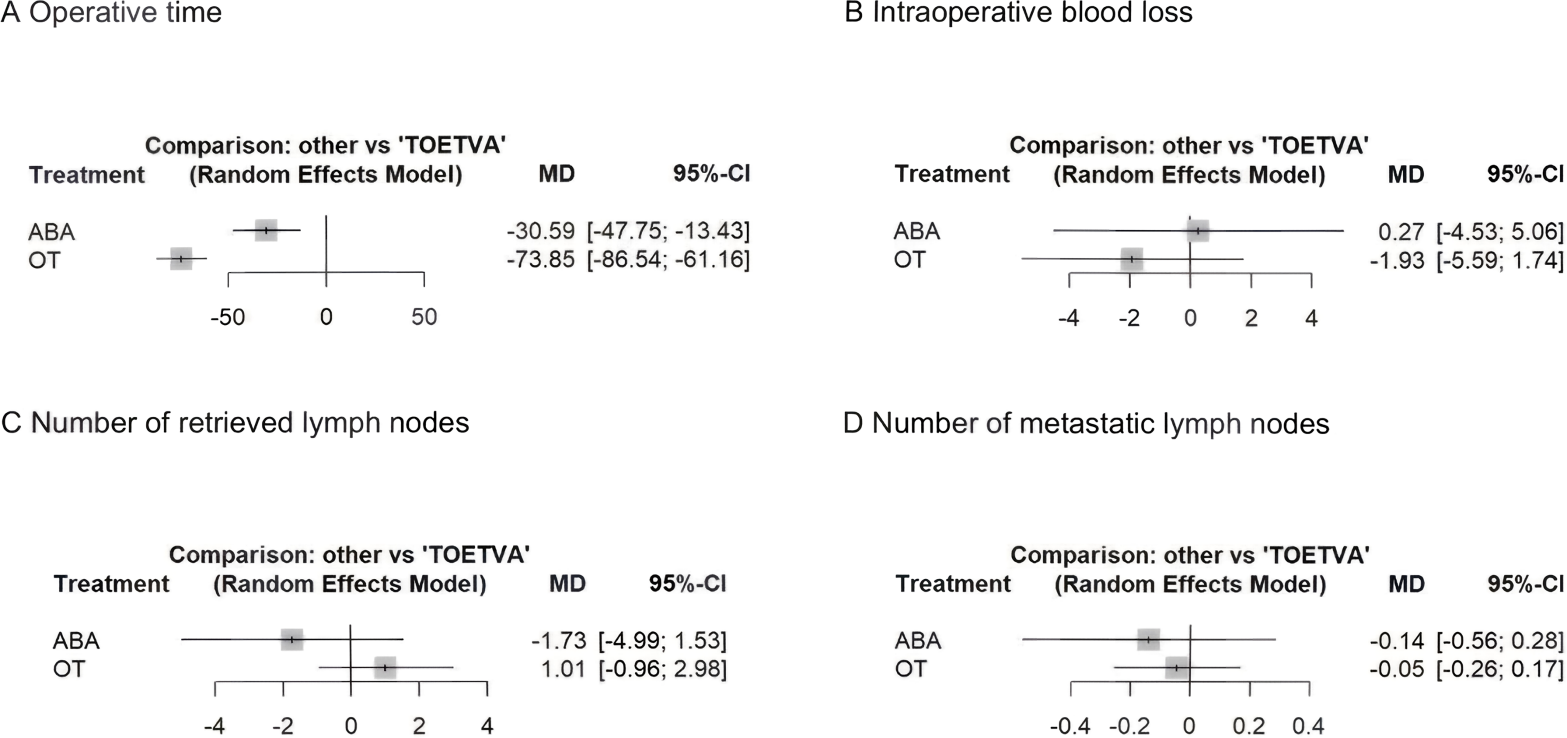
Network meta-analysis results for intraoperative outcomes. Standardized mean differences (SMD) with 95% confidence intervals (CI) containing a value of 0 were considered insignificant. TOETVA, transoral endoscopic thyroidectomy vestibular approach; OT, open thyroidectomy; ABA, endoscopic thyroidectomy via the axillary breast approach; vs., versus. **(A)**, Forest plot shows comparison of the operative time in minutes. **(B)**, Forest plot shows comparison of the intraoperative blood loss in ml. **(C)**, Forest plot shows comparison of the number of retrieved lymph nodes. **(D)**, Forest plot shows comparison of the number of metastatic lymph nodes.

The network meta-analysis results of intraoperative outcomes are shown in **Figures 2A–D**. Twenty-six studies, including 4889 patients, reported operative time. TOETVA was associated with significantly longer operative time compared with OT (MD = 73.85; 95% CI: 61.16–86.54) and ABA (MD = 30.59; 95% CI: 13.43–47.75). Twelve studies reported intraoperative blood loss, with no significant differences among the three approaches (all P > 0.05). Fifteen studies reported the number of retrieved central lymph nodes, and 13 reported metastatic lymph nodes, no significant differences were found for either outcome (all P > 0.05), indicating comparable completeness of lymph node dissection.

#### 3.3.2 Postoperative outcomes

**Figure 3:**
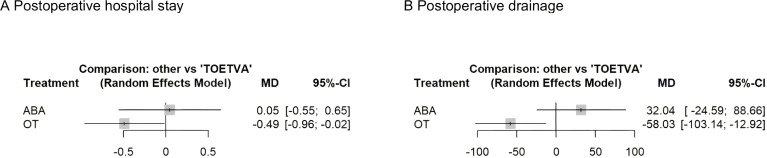
Network meta-analysis results for postoperative outcomes. Standardized mean differences (SMD) with 95% confidence intervals (CI) containing a value of 0 were considered insignificant. TOETVA, transoral endoscopic thyroidectomy vestibular approach; OT, open thyroidectomy; ABA, endoscopic thyroidectomy via the axillary breast approach; vs., versus. **(A)**, Forest plot shows comparison of the postoperative hospital stay in days. **(B)**, Forest plot shows comparison of the amount of postoperative drainage in ml.

The network meta-analysis results of postoperative outcomes are shown in **Figures 3A, B**. Twenty-two studies reported data of postoperative hospital stay. OT had a significantly shorter stay than TOETVA (MD = 0.49; 95% CI: 0.02–0.96), and the other comparisons were not significant. Regarding postoperative drainage volume, OT had significantly less drainage than TOETVA (MD = 58.03, 95% CI: 12.92 to 103.14, P = 0.012) and ABA (MD = 90.06, 95% CI: 36.69 to 143.44, P < 0.001). TOETVA showed less drainage than ABA, but the difference was not statistically significant (MD = 32.04, 95% CI: −24.59 to 88.66, P = 0.268).

#### 3.3.3 Postoperative complications

**Figure 4:**
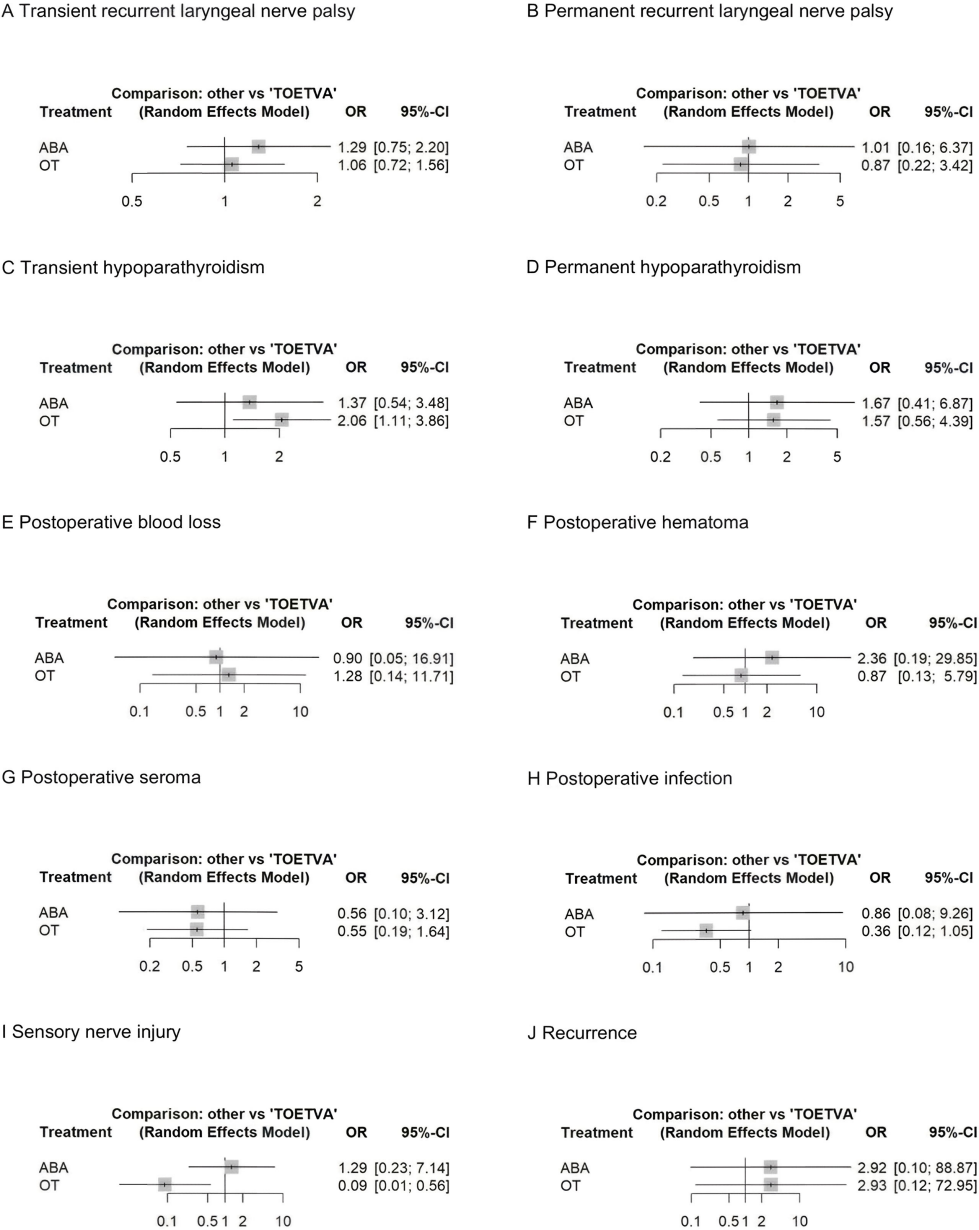
Network meta-analysis results for postoperative complications and recurrence. Odds ratios (OR) with 95% confidence intervals (CI) containing a value of 1 were considered insignificant. TOETVA, transoral endoscopic thyroidectomy vestibular approach; OT, open thyroidectomy; ABA, endoscopic thyroidectomy via the axillary breast approach; vs., versus. **(A)**, Forest plot shows comparison of transient recurrent laryngeal nerve palsy. **(B)**, Forest plot shows comparison of permanent recurrent laryngeal nerve palsy. **(C)**, Forest plot shows comparison of transient hypoparathyroidism. **(D)**, Forest plot shows comparison of permanent hypoparathyroidism. **(E)**, Forest plot shows comparison of postoperative blood loss. **(F)**, Forest plot shows comparison of postoperative hematoma. **(G)**, Forest plot shows comparison of postoperative seroma. **(H)**, Forest plot shows comparison of postoperative infection. **(I)**, Forest plot shows comparison of sensory nerve injury. **(J)**, Forest plot shows comparison of recurrence.

The network meta-analysis results of postoperative outcomes are shown in **Figures 4A–J**. No significant differences were found among TOETVA, OT, and ABA regarding temporary or permanent RLN injury. For hypoparathyroidism, no significant differences were observed for the permanent form. However, TOETVA was superior to OT for transient hypoparathyroidism (OR = 0.48; 95% CI: 0.26–0.90; P = 0.023). Seven studies reported sensory nerve injury. OT was associated with a significantly lower risk than TOETVA (OR = 0.09; 95% CI: 0.01–0.56; P = 0.010), while no significant difference was observed between ABA and TOETVA. Regarding other complications, no significant differences were found among the three approaches for postoperative blood loss, hematoma, seroma, infection, or recurrence (all P > 0.05).

### 3.4 Ranking hierarchy analysis

**Figure 5:**
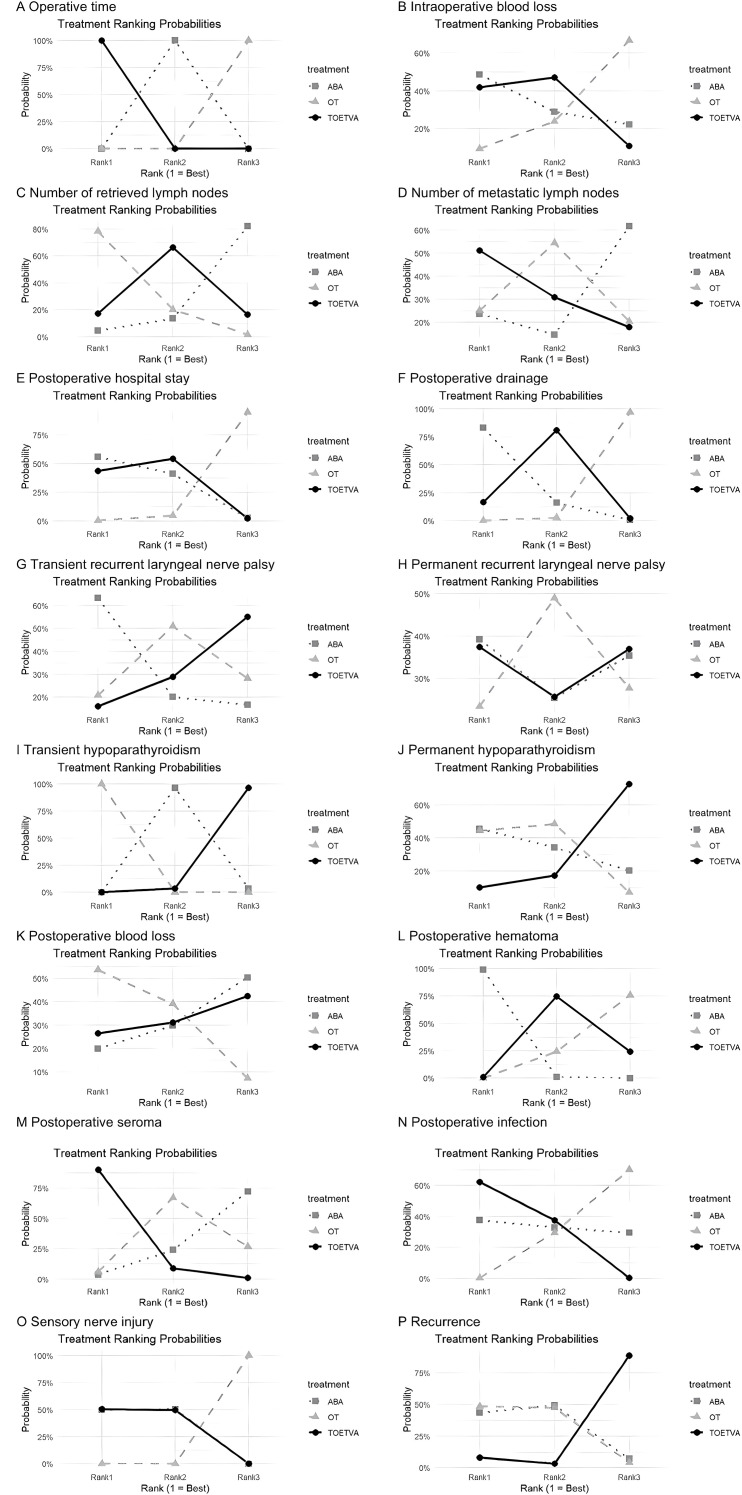
Network meta-analysis results for ranked probabilities of approaches. TOETVA, transoral endoscopic thyroidectomy vestibular approach; OT, open thyroidectomy; ABA, endoscopic thyroidectomy via the axillary breast approach. **(A)**, line graph shows the treatment ranking probabilities for operative time. **(B)**, line graph shows the treatment ranking probabilities for intraoperative blood loss. **(C)**, line graph shows the treatment ranking probabilities for the number of retrieved lymph nodes. **(D)**, line graph shows the treatment ranking probabilities for the number of metastatic lymph nodes. **(E)**, line graph shows the treatment ranking probabilities for the postoperative hospital stay in days. **(F)**, line graph shows the treatment ranking probabilities for the amount of postoperative drainage in ml. **(G)**, line graph shows the treatment ranking probabilities for transient recurrent laryngeal nerve palsy. **(H)**, line graph shows the treatment ranking probabilities for permanent recurrent laryngeal nerve palsy. **(I)**, line graph shows the treatment ranking probabilities for transient hypoparathyroidism. **(J)**, line graph shows the treatment ranking probabilities for permanent hypoparathyroidism. **(K)**, line graph shows the treatment ranking probabilities for postoperative blood loss. **(L)**, line graph shows the treatment ranking probabilities for postoperative hematoma. **(M)**, line graph shows the treatment ranking probabilities for postoperative seroma. **(N)**, line graph shows the treatment ranking probabilities for postoperative infection. **(O)**, line graph shows the treatment ranking probabilities for sensory nerve injury. **(P)**, line graph shows the treatment ranking probabilities for recurrence.

The ranking probabilities of the three approaches are presented in **Figure 5**. OT ranked best for operative time, intraoperative blood loss, the number of retrieved lymph nodes, postoperative hospital stay, and sensory nerve injury, but it ranked worst for postoperative blood loss. TOETVA ranked best for the number of metastatic lymph nodes, transient and permanent RLN palsy, transient and permanent hypoparathyroidism, and recurrence, but worst for postoperative seroma and infection. ABA ranked worst for intraoperative blood loss, postoperative hospital stay, drainage, transient and permanent RLN palsy, and hematoma.

## 4 Discussion

This network meta-analysis is the first to comprehensively compare the efficacy and safety of TOETVA, OT, and ABA for patients with PTC across 16 clinically relevant outcomes. Our findings demonstrate that while TOETVA is associated with longer operative times and a longer postoperative hospital stay compared to OT, it offers comparable oncological radicality, a lower risk of permanent hypoparathyroidism. Importantly, no significant differences were observed in the rates of RLN injury, postoperative blood loss, hematoma, seroma, infection, or recurrence among the three approaches. These results support TOETVA as a safe and feasible alternative to conventional approaches in carefully selected patients with PTC, provided the surgeon has undergone appropriate training.

The most critical finding of this analysis is the equivalence among TOETVA, OT, and ABA in terms of the number of central lymph nodes dissected, the number of positive lymph nodes, and the postoperative recurrence rate. Achieving complete tumor resection and adequate lymph node dissection is the fundamental requirement of any oncological procedure. The available evidence suggests comparable oncologic outcomes among these approaches. This contradicts early concerns that the endoscopic view or the working angle of TOETVA might limit the completeness of central compartment dissection (43). The magnified visualization afforded by endoscopic systems may, in fact, facilitate meticulous dissection, potentially offsetting the challenges posed by OT. This oncological equivalence is the cornerstone supporting the clinical adoption of TOETVA as a valid alternative to OT for selected PTC patients.

The finding that TOETVA requires a significantly longer operative time than OT is consistent with the literature (44). Several factors contribute to this: the need for port placement in the narrow oral vestibule, creation of a subcutaneous tunnel from the oral cavity to the thyroid bed, establishment of sufficient working space with CO_2_ insufflation, and the inherent technical demands of endoscopic instrumentation in a confined, inverted anatomy (25). Importantly, operative time is highly dependent on surgical experience. Multiple studies have documented a significant reduction in TOETVA operative time after the initial 30–50 cases, indicating a steep learning curve (45, 46). As TOETVA becomes more widely adopted and surgeons advance along the learning curve, this time gap is expected to narrow (47).

Regarding postoperative recovery, OT was associated with significantly shorter hospital stays and lower drainage volumes than TOETVA. Although the absolute difference in hospital stay was modest, the extended recovery following TOETVA may be attributed to the following factors. First, incision classification: TOETVA involves an intraoral incision, making it a clean-contaminated procedure (Class II), whereas OT is classified as a clean procedure (Class I) (48). Consequently, surgeons may adopt a more cautious postoperative monitoring strategy for TOETVA patients, including observation for potential infectious complications. Second, subcutaneous dissection: TOETVA requires extensive flap creation, which can induce greater local inflammation and serous fluid accumulation, leading to increased drainage output. Prolonged drainage may be maintained to prevent seroma formation, thereby potentially extending hospital stay. Notably, no significant differences in hospital stay or drainage volume were observed between ABA and TOETVA, suggesting that the two endoscopic approaches share similar postoperative recovery profiles, both involving more extensive tissue manipulation than OT.

In terms of postoperative complications, TOETVA was superior to OT in reducing the risk of transient hypoparathyroidism (OR = 0.48). The observed advantage may be explained by the enhanced visualization provided by the endoscopic approach. Regarding permanent hypoparathyroidism, no statistically significant difference was observed among the three groups. Nevertheless, the SUCRA ranking analysis indicated that TOETVA had the highest probability of being the optimal approach for preventing this complication. Permanent hypoparathyroidism necessitates lifelong calcium and vitamin D supplementation, which is associated with an increased risk of renal impairment and a diminished quality of life. Consequently, despite the absence of statistical significance in the pairwise comparisons, this ranking advantage may hold clinical relevance. Notably, the low overall event rates across all groups likely limited the statistical power to detect modest differences, suggesting that larger prospective studies are warranted to confirm this observed trend.

The three approaches demonstrated equivalent rates of temporary and permanent RLN injury. The equivalence suggests that, with routine intraoperative nerve monitoring and meticulous technique, the risk to the RLN is comparable regardless of the access route (49).

In contrast, sensory nerve injury was significantly more common in TOETVA and ABA compared to OT. Sensory nerve injury was reported in seven studies, 22 cases occurred among 309 patients in the TOETVA group, 35 cases among 185 patients in the ABA group, and only 2 cases among 376 patients in the OT group. But it should be noted that the definition of this complication was not entirely consistent across these studies. For TOETVA, sensory nerve injury was primarily manifested as lower lip numbness, perioral numbness, and chin region hypoesthesia, which correspond to mental nerve injury. In contrast, for ABA, sensory nerve injury included anterior chest wall paresthesia, infraclavicular numbness, and cervical plexus sensory branch irritation, reflecting the extensive subcutaneous flap dissection required for this approach. For OT, sensory nerve injury was reported as paresthesia in the surgical area and chin region. Both TOETVA and ABA were associated with significantly higher incidences of sensory nerve injury compared with OT. In TOETVA, the lateral incisions are often placed near the mental foramen, and the subsequent instrumentation can stretch or directly injure the nerve (50). In ABA, port insertion and dissection in the upper chest and axilla can similarly affect the sensory branches of the cervical plexus. Whereas no significant difference was detected between TOETVA and ABA, regarding sensory nerve injury, this finding should be interpreted with caution. The lack of a significant difference may be attributable to several factors. First, the relatively small sample size of studies directly comparing TOETVA with ABA may have limited the statistical power to detect a true difference. Second, the included studies exhibited considerable heterogeneity in their definitions and assessment methods for sensory nerve injury. These disparate observation perspectives and judgment criteria for what constitutes a sensory nerve injury event may have introduced bias and obscured a true underlying difference between the two endoscopic approaches. Consequently, the observed absence of a significant difference may not fully reflect the true clinical reality. Larger-scale prospective cohort studies with standardized outcome definitions and validated assessment tools are warranted to elucidate the genuine difference in sensory nerve injury between TOETVA and ABA. While usually temporary and of low morbidity, sensory nerve injury is approach-specific and should be explicitly discussed during preoperative consent for endoscopic thyroidectomy. To mitigate this risk, careful placement of lateral incisions and gentle retraction are recommended (51).

No significant differences were found among the three approaches for postoperative blood loss, hematoma, seroma, or infection. However, the comparison between OT and TOETVA for postoperative infection approached statistical significance (P = 0.060), suggesting a potential trend toward increased infectious risk with TOETVA. As noted, a plausible explanation for this observation is that TOETVA requires an intraoral incision, thereby converting a conventional Class I (clean) incision into a Class II (clean-contaminated) incision. Most protocols for TOETVA include perioperative prophylactic antibiotics (e.g., broad-spectrum coverage or a second-generation cephalosporin), but robust evidence guiding the selection, timing, and duration of antibiotics is lacking (48). This represents a key knowledge gap warranting future prospective randomized trials.

Furthermore, this analysis was unable to evaluate several approach-specific complications due to sparse reporting, including CO_2_ embolism, flap perforation, chin depression, and subcutaneous emphysema (48, 52, 53). As TOETVA is a relatively novel technique, the true incidence of these events may be underreported but is expected to decline with greater experience and standardized protocols.

Several limitations of this network meta-analysis must be acknowledged. First, all included studies were non-randomized observational designs (retrospective or prospective cohorts), rendering them susceptible to selection bias and residual confounding. Second, the sample sizes for comparisons involving ABA were relatively small, limiting statistical power for detecting modest differences and increasing the risk of type II error. Third, substantial clinical heterogeneity was present across studies regarding surgeon experience, patient selection criteria, extent of thyroidectomy, and perioperative management. Fourth, follow-up durations varied considerably, which may affect the accuracy of recurrence rate estimates, particularly for delayed locoregional recurrence. Finally, publication bias cannot be excluded; studies with negative or null results may be underreported.

This network meta-analysis has several implications for clinical practice and future research. For appropriately selected patients with low- intermediate-risk PTC (e.g., tumors ≤2 cm, no extensive extrathyroidal extension, no bulky central lymphadenopathy) (54), TOETVA represents a safe and effective alternative to OT, particularly for patients who place a high value on scarless cosmesis. Surgeons adopting TOETVA should undergo structured training, including cadaveric or simulation-based practice, to overcome the learning curve and minimize operative time and complications (55). Future research should focus on: (1) large-scale, multicenter randomized controlled trials comparing TOETVA and OT with long-term oncologic follow-up (≥5 years); (2) standardized reporting of approach-specific complications (e.g., CO_2_ embolism, mental nerve injury); (3) patient-reported outcome measures (PROMs), including cosmetic satisfaction, quality of life, and body image; (4) delineation of optimal perioperative antibiotic protocols for TOETVA.

In conclusion, this network meta-analysis demonstrates that TOETVA, OT, and ABA offer comparable oncological efficacy and overall safety for the treatment of PTC. OT remains the gold standard in terms of shortest operative time, fastest postoperative recovery, and best mental nerve protection. TOETVA, while requiring the longest operative time, offers the distinct advantages of superior cosmesis and a lower risk of permanent hypoparathyroidism. ABA occupies an intermediate position. Despite a potential but statistically non-significant trend toward increased infection risk, the available evidence supports TOETVA as a safe and feasible alternative to conventional thyroidectomy in carefully selected patients with PTC. High-quality, large-scale randomized controlled trials with long-term follow-up are warranted to confirm these findings and further refine patient selection and perioperative protocols.
