# Supplementary material for: Efficacy and safety of transoral endoscopic thyroidectomy vestibular approach of papillary thyroid carcinoma: a network meta-analysis
Source: Front Oncol. 2026 Jul 1;16:1869610. doi: 10.3389/fonc.2026.1869610 (PMC13368631; doi:10.3389/fonc.2026.1869610)
Supplement: Supplementary file 1 [file DataSheet1.pdf]

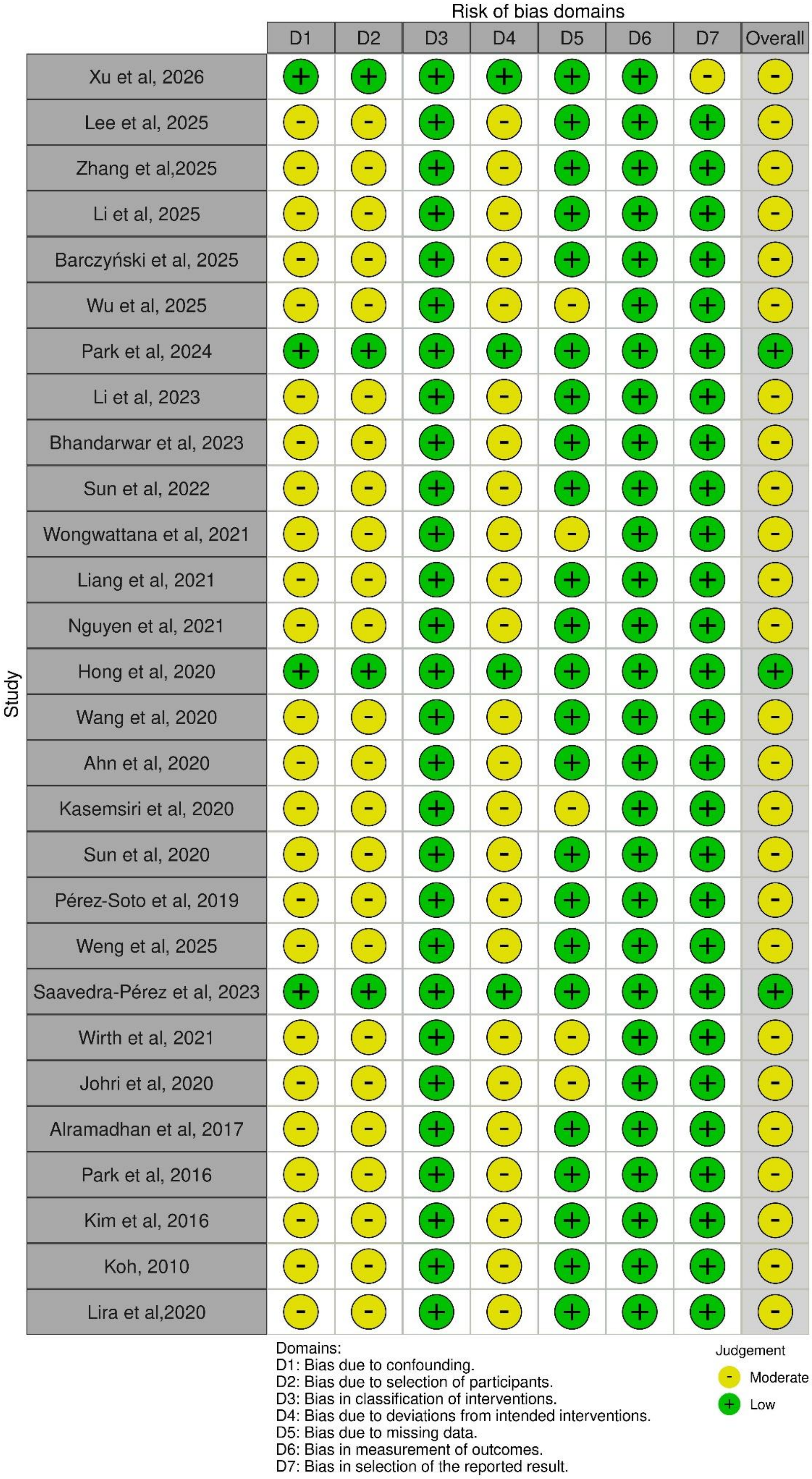

Figure S1. The results of the quality assessment using the ROBINS-I tool.

A Operative time

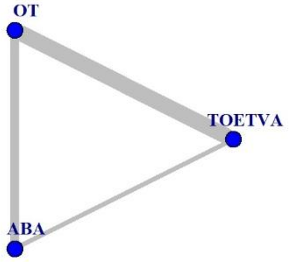

B Intraoperative blood loss

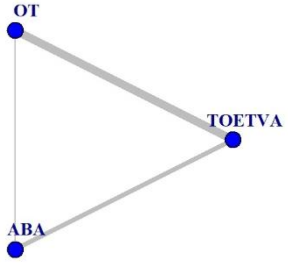

C Number of retrieved lymph nodes

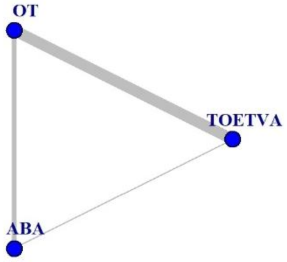

D Number of metastatic lymph nodes

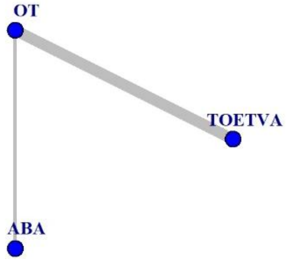

E Postoperative hospital stay

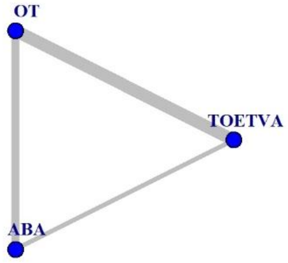

F Postoperative drainage

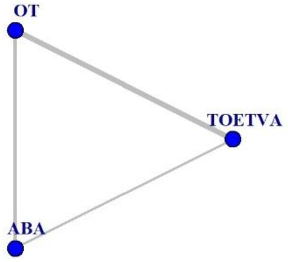

G Transient recurrent laryngeal nerve palsy

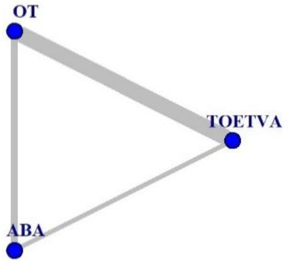

H Permanent recurrent laryngeal nerve palsy

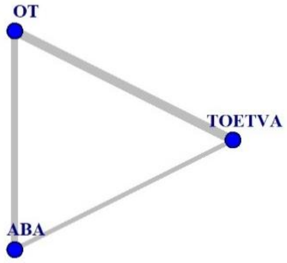

I Transient hypoparathyroidism

J Permanent hypoparathyroidism

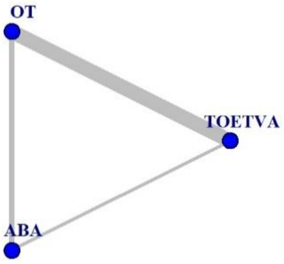

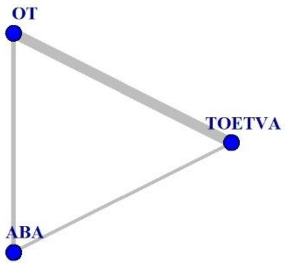

K Postoperative blood loss

L Postoperative hematoma

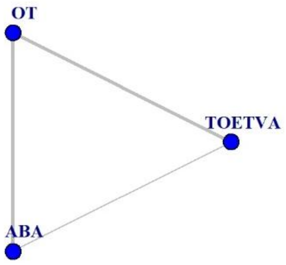

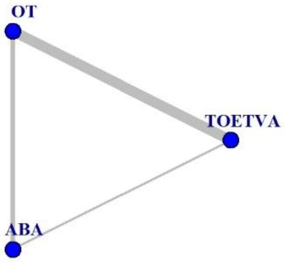

M Postoperative seroma

N Postoperative infection

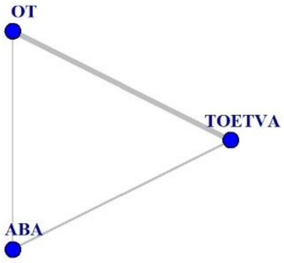

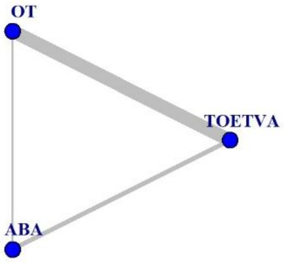

O Sensory nerve injury

J Recurrence

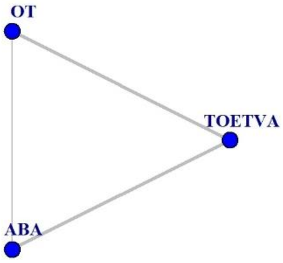

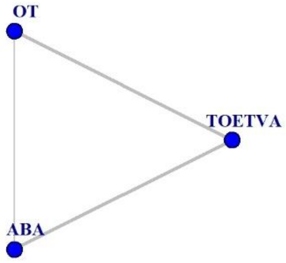

Figure S2. Network diagrams of relationships among different approaches. The thickness of each edge presents the number of studies, specified on the edge, comparing the linked approaches. OT, open thyroidectomy; ABA, endoscopic thyroidectomy via the axillary breast approach.
